# Residual gait deviations in children treated by medial open reduction for developmental dysplasia of the hip at long-term follow-up: a comparison with healthy controls

**DOI:** 10.1007/s00264-024-06263-9

**Published:** 2024-08-07

**Authors:** Mehmet Demirel, Halenur Evrendilek, N. Ekin Akalan, Fuat Bilgili, Emre Meriç, Shavkat Kuchimov, Kübra Önerge

**Affiliations:** 1 Department of Orthopaedics and Traumatology, İstanbul School of Medicine, İstanbul University, Istanbul, Turkey; 2 Physiotherapy and Rehabilitation Department, Faculty of Health Sciences, İstanbul Kültür University, Istanbul, Turkey; 3 Institute of Biomedical Engineering, Boğaziçi University, Istanbul, Turkey

**Keywords:** Developmental dysplasia of the hip, Medial open reduction, Gait analysis, Stiff-knee gait, Residual gait deviations

## Abstract

**Purpose:**

This study aimed to analyze and compare gait patterns and deviations at long-term follow-up in children who received medial open reduction (MOR) before 18 months for unilateral or bilateral hip developmental dysplasia (DDH).

**Methods:**

A retrospective chart review was conducted on children who underwent MOR. The study population was divided into two groups: the unilateral group, including unilateral (five children with unilateral) and bilateral (five children with bilateral DDH). Ten healthy children were recruited for the control group. Spatiotemporal, kinematic, stiff-knee gait (SKG), and kinetic gait characteristics were analyzed.

**Results:**

Stance time was significantly shorter in both the unilateral (median [IQR]; 590 ms, [560.0–612.5] and bilateral (575 ms, [550–637.5]) groups than in the control group (650, [602.5–677.5]) (*p* < 0.001), whereas swing time did not differ substantially (*p* = 0.065) There was no considerable difference in the mean knee flexion at swing between the unilateral (31.6°, [30–36]) and control (30.11°, [27.8–33.6] groups (*p* > 0.05), but the bilateral group (28.5°, [24.9–32.1]) showed the lower values than the other groups (*p* < 0.001 for bilateral vs unilateral group; *p* = 0.008 bilateral vs unilateral group). All the SKG parameters significantly differed among the groups in multi-group comparisons (*p* < 0.001 for each parameter). Three children had borderline SKG, and two had not-stiff limbs in the unilateral group. In the bilateral group, four children had stiff limbs, and one had borderline SKG. Most kinetic gait parameters were not statistically different between groups (*p* > 0.05).

**Conclusion:**

This study has revealed notable deviations in gait patterns of children with DDH treated by MOR at long-term follow-up compared to healthy children’s gait. MOR could negatively affect pelvic motion during gait due to impaired functions of the iliopsoas and adductor muscles, and SKG can be encountered secondary to iliopsoas weakness.

## Introduction

The main goal of the treatment for developmental dysplasia of the hip (DDH) is to obtain a concentric stable reduction of the femoral head into the acetabulum and ensure proper development of all the hip structures [1]. To accomplish this goal, the initial treatment is typically non-operative with Pavlik Harness within the first six months of life [2, 3]. Open reduction is generally reserved for children aged six to 18 months in whom non-operative methods fail to obtain a stable reduction. Many authors suggest open reduction as the first-line treatment in children older than 18 months [4–6]. The two main surgical approaches for open reduction are medially based approach open reductions and anterior approach open reductions. The medial open reduction (MOR) is an accepted technique that can permit direct access to all the major obstacles to concentric reduction, leaving the hip abductors or iliac apophysis intact [7].

The iliopsoas and hip adductor tendons (particularly adductor longus) constitute the main extra-articular soft tissue obstacles to reducing the femoral head into the acetabulum. Accordingly, the iliopsoas tendon is routinely released from the lesser trochanter during both MOR and anterior open reduction to facilitate hip exposure and remove a potential obstacle to reduction [7, 8]. Whenever necessary to enlarge the safe zone of abduction, the adductor longus tendon is typically sectioned (tenotomy). The adductor tenotomy is performed percutaneously or by a medial mini incision over the adductor muscles in anterior open reduction, while it can be done openly at its origin via the same approach during MOR [9, 10]. In contrast, the adductor muscle group (adductor longus/brevis/magnus and gracilis) and iliopsoas muscle play a vital role in gait and walking performance; thus, sectioning these tendons may result in hip muscle weakness and a possible gait disturbance in children with DDH at long-term. Furthermore, weakening iliopsoas muscle has been recently reported to induce a pattern of stiff-knee gait (SKG) characterized by reduced or delayed peak knee flexion (PKF) during the swing phase of the gait cycle by reducing the hip flexion velocity even in healthy participants [11].

According to our literature review, only a few studies have examined gait patterns in children surgically treated by open hip reduction with soft tissue release [7, 12–15]. Moreover, the available data were either obtained from sagittal-plane kinetics and kinematics of the hip joint [7, 12] or limited to gait analyses of children with unilateral DDH [13, 15]. No study has specifically examined 3-D gait patterns in children with bilateral DDH treated by MOR. To our knowledge, there needs to be more literature on the differences in gait parameters between children treated by unilateral versus bilateral MOR. In our opinion, unilateral adductor and iliopsoas tenotomy may influence pelvic motion. Furthermore, iliopsoas tenotomy could influence swing-phase knee flexion and contribute to SKG in children treated by MOR.

This study aimed to analyse and compare gait patterns and deviations at long-term follow-up in children who underwent MOR with the soft-tissue release, including the iliopsoas and adductor longus tenotomies, before 18 months for either unilateral or bilateral DDH. Our first hypothesis was that children with DDH treated by MOR could develop significant gait deviations in the long-term compared to healthy controls due to impaired functions of the iliopsoas and adductor muscles. Our second hypothesis was that MOR could negatively affect pelvic motion during the gait cycle of the affected extremity due to impaired functions of the iliopsoas and adductor muscles. Our third hypothesis was that sagittal-plane knee kinematics might alter due to long-term weakness in the iliopsoas muscle and lead to SKG in children with DDH undergoing MOR.

## Patients and methods

A retrospective chart review was conducted on children who underwent MOR to treat DDH between the years 2004 and 2011 at our institution. The inclusion criteria for the study were: I) a diagnosis of idiopathic DDH, II) radiographic DDH severity of Tönnis grade 3 to 4 [16], III) treated surgically by unilateral or bilateral MOR in one stage before the age of 18 months, IV) complete medical and radiographic records, V) a minimum of five years of age at the time of gait analysis, VI) no redislocation or residual acetabular dysplasia, VII) favourable radiographic outcome at the final follow-up as per the Severin score (type I or type II) [17], and VI) being willing to participate in the study. The exclusion criteria included: I) lost to follow-up, II) inadequate medical record, III) concomitant neuromuscular comorbidities, VI) previous failed MOR, and V) unwilling to participate in the study.

Thirteen children were evaluated based on the above eligibility criteria in the DDH group. After excluding three children (one was lost to follow-up, one had inadequate medical record, and one had previously failed MOR), the remaining ten children (9 girls, one boy; median age at final follow-up = 11 years [interquartile range {IQR} = 11–13.5]) were included in the study population. The study population was then divided into two groups: the unilateral group, including five children with unilateral DDH (5 girls; median age at final follow-up = 12 years, [IQR = 11.5 – 15]) and the bilateral group, including five children with bilateral DDH (4 girls, one boy; median age at final follow-up = 11 years, [IQR = 9–13.5]). The median age at the time of operation was eight months [IQR = 7 – 15]) in the unilateral group and nine months [IQR = 6–18]) in the bilateral group. The median overall follow-up was 150 months [IQR = 80 – 192]. The median follow-up was 159 months [IQR = 129 – 192] in the unilateral group and 140 months [IQR = 80–172]) in the bilateral group (Table 1).

**Table 1:** Demographic characteristics of the study participants

Variables	Overall Study Population (*n* = 10 children)	Unilateral group (*n* = 5 children)	Bilateral group (*n* = 5 children)	Control group (*n* = 10 children)
Age at the time of surgery (months)		8 (7 – 15)	9 (6 – 18)	–
Age at the final follow-up (years)	11 (11–13.5)	12 (11.5 – 15)	11 (9–13.5)	11 (11 – 13)
Gender				
• Girl		5	4	7
• Boy		–	1	3
Follow-up (months)	150 (80 – 192)	159 (129–192)	140 (80–172)	–

Data are presented as median (IQR)

*Children undergoing medial open reduction before 18 months for unilateral or bilateral developmental dysplasia of the hip

**Fig. 1:**
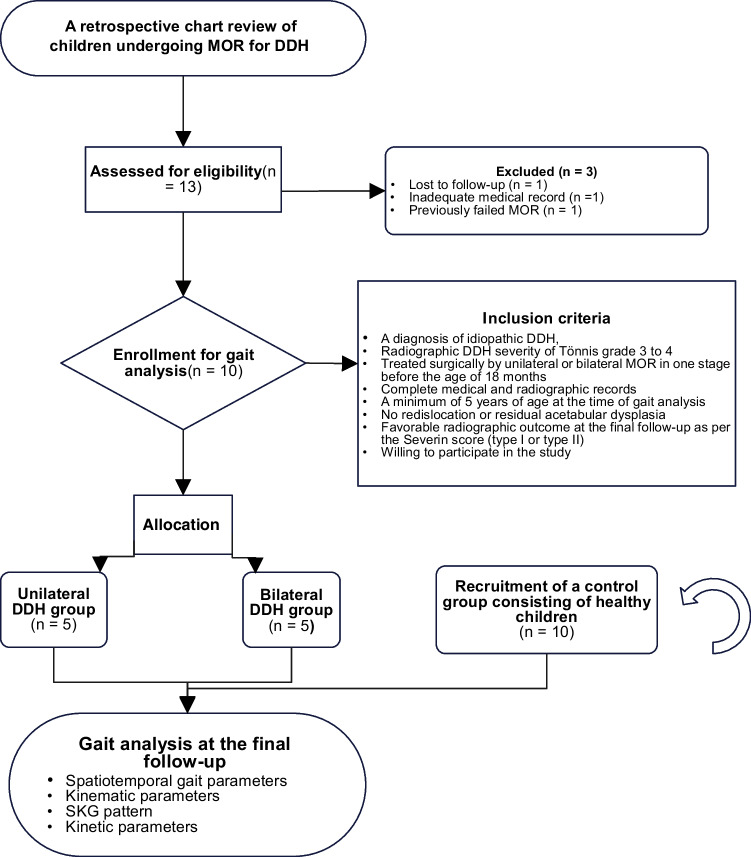
Flow diagram of the study participants

A convenience sample of ten healthy children (20 lower extremity, seven girls, three boys; median age = 11 years, [IQR = 11–13]) were recruited for the control group. Children in this group were volunteers from the surrounding community of our hospital who had no known orthopaedic abnormalities that affected their gait. Participants in each group had similar age and sex distribution (Table 1). Parents were informed that medical records could be used for only scientific purposes; written informed consent was obtained. The study protocol was approved by our institutional ethical committee on human research and was carried out per the Declaration of Helsinki guidelines. A flow diagram of study participants is shown in Fig. 1.

## Gait analysis

**Fig. 2:**
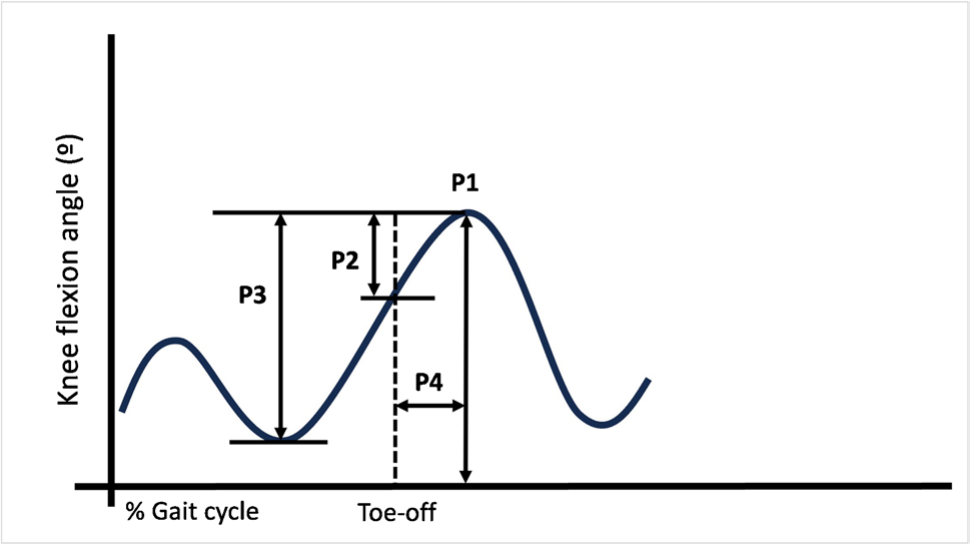
The four kinematic gait parameters chosen as the criteria of whether a patient walked with an SKG: P1) peak knee flexion angle, P2) range of knee flexion in early swing between toe-off and peak knee flexion, P3) total range of knee flexion, and P4) peak knee flexion time in the swing. A parameter was indicative of SKG if the value was more than two standard deviations below the average normal value in the case of parameters 1–3 or more than two standard deviations above the average normal value in the case of parameter 4. A limb was considered ‘‘stiff’’ if three or more of the parameters were indicative of SKG. A limb was considered ‘‘not-stiff’’ if one or none of the parameters were indicative of SKG. If two of the parameters were indicative of SKG, the limb was classified as a borderline case

Gait analysis was conducted using an optoelectronic system with six cameras and two force plates (ELITE 2002; BTS Bioengineering, Milan, Italy) between January 2019 and May 2019 in the Gait Analysis Laboratory of our university according to the description of Davis et al. (modified Helen Hayes) [18]. Each child was asked to walk with their natural gait along an 8-m walkway, and the following gait parameters were collected from the involved and uninvolved sides under the supervision of an experienced, specially trained paediatric physiotherapist and compared to those of healthy participants:

- *Spatiotemporal gait parameters*: velocity of gait (m/s), stance time (sec), swing time (sec), step length (m), and stride length (m).
- *Kinematic parameters*: pelvic tilt (°), pelvis obliquity (°), pelvic rotation (°), hip flexion/extension (°), hip abduction/adduction (°), hip rotation (°), knee flexion/extension (°), knee valgus/varus (°), ankle dorsiflexion/plantar flexion (°).
- *SKG pattern:* four kinematic gait parameters were chosen as the criteria to determine the SKG pattern based on the SKG definition of Goldberg et al., consisting of P1) PKF angle, P2) range of knee flexion between toe-off and PKF, P3) total range of knee flexion, and P4) PKF in swing from toe-off to PKF [19]. For each study participant from the unilateral or bilateral group, a parameter was indicative of SKG if the value was more than two standard deviations below the average normal value in the case of parameters 1–3 or more than two standard deviations above the average normal value in the case of parameter 4. A limb was considered ‘‘stiff’’ if three or more of the parameters were indicative of SKG. A limb was considered ‘‘not-stiff’’ if one or none of the parameters were indicative of SKG. If two of the parameters were indicative of SKG, the limb was classified as a borderline case [19] (Fig. 2). The average normal value for each SKG parameter was calculated based on the data obtained from the control group. The mean of each SKG parameter obtained from both lower extremities was considered for the SKG analysis in the bilateral group.
- *Kinetic parameters:* moment and powers of hip, knee, and ankle in the sagittal plane.

## Management and follow-up protocol

Open reduction was performed using the Ferguson medial approach, as previously described [20] in the literature, by the same surgical team before 18 months. In all children, adductor longus and iliopsoas tenotomies were performed. Children with bilateral DDH were operated on in the same session. After a concentric hip reduction was obtained, all children were placed in a hip spica cast postoperatively, with hip abduction of < 60° and flexion of not > 100° for three months based on the surgeon’s preference. The cast was routinely changed at least once under general anaesthesia at the midpoint of this period. Patients were then put in an abduction orthosis with both hips flexed at 90° and abducted at 30° for nearly 12 weeks (full-time for the first six weeks and only during sleep for the next six weeks). No specific rehabilitation program was performed.

## Statistical analysis

Data management and analysis were performed using SPSS 25.0 (IBM, Armonk, New York, USA), and statistical significance was set at *p* < 0.05. Normality tests were conducted using the Shapiro–Wilk test. Data are presented as ‘’median (IQR)’’ or ‘’mean and standard deviation’’. The Kruskal–Wallis test was used to analyse the difference between DDH and control groups, and then the Mann–Whitney U test was used to compare the two groups. Significance levels for multiple tests were adjusted by the Bonferroni–Dunn method.

## Results

No statistically significant differences were found in demographic characteristics between groups (*p* < 0.05 for each variable) (Table 1).

## Spatiotemporal gait parameters

Table 2 summarizes the results of spatiotemporal gait parameters. The mean velocity was similar among the groups (*p* = 0.94). Stance time was significantly shorter in both the unilateral and bilateral groups than in the control group, whereas swing time did not differ substantially. While anterior step length and double support time were significantly shorter in the bilateral group than in the control group, no significant differences in those parameters were observed between the unilateral and bilateral groups. Step width was wider in the unilateral group than in the bilateral group, and stride length was significantly shorter in the bilateral group than in the control group.

**Table 2:** Spatiotemporal gait parameters

Variables	Unilateral group (*n *= 5)	Bilateral group (*n* = 5)	Control group (*n* = 10)	Multi-group comparison (*p* values)	Pair-wise group comparison (*p* values)
Velocity (m/s)	1.1 (1.1–1.2)	1.1 (1–1.2)	1.1 (1–1.2)	0.94	—
Stance time (msec)	590.0 (560.0–612.5)	575.0 (550.0–637.5)	650.0 (602.5–677.5)	< 0.001*	Bi vs C < 0.001* Uni vs C < 0.001*
Swing time (msec)	460.0 (400.0–482.5)	410.0 (380.0–475.5)	430.0 (392.5–450.0)	0.065	—
Double sup. Time (msec)	90.0 (65.0–120.0)	90.0 (62.5–110.0)	109.0 (100.0–120.0)	0.001*	Bi vs C < 0.001* Uni vs C < 0.001*
Cadence (step/min)	113.5 (109.0–124.3)	120.5 (111.8–128.8)	112.5 (105.8–117.3)	0.04*	Bi vs C 0.015* Uni vs C 0.018*
Step width (mm)	111.5 (92.0–151.5)	95.5 (81.8–109.0)	100.5 (80.5–112.5)	0.027*	Bi vs Uni 0.013* Uni vs C 0.015*
Stride length (mm)	1204.0 (1113.5–1336.0)	1167.5 (1187.3–1245.7)	1253.5 (1182.3–1321.0)	0.001*	Bi vs C < 0.001*
Anterior Step length (mm)	626.5 (563.5–666.5)	583.5 (519.3–625.3)	623.5 (595.5–670.8)	0.001*	Bi vs C < 0.001*

Data are presented as median (IQR). In pair-wise group comparisons, only statistically significant associations are presented

^*^Statistical significance was set at *p* < 0.05

## Kinematic gait analysis

Table 3 outlines the results of kinematic gait parameters. The mean anterior pelvic tilt was lower in both unilateral and bilateral groups than in the control group, while there was no significant difference in pelvic rotation among the groups. The pelvic obliquity range was significantly higher in the unilateral group than in the bilateral group. Hip ranges at sagittal and frontal planes were lower in both unilateral and bilateral DDH groups than in the control group. Hip internal rotation significantly differed among the groups, which was higher in the bilateral group compared to unilateral and control groups.

**Table 3:** Kinematic gait parameters

Variables	Unilateral group (*n* = 5)	Bilateral group (*n* = 5)	Control group (*n* = 10)	Multi group comparison (*p* values)	Pairwise group comparison (*p* values)
Mean pelvic tilt (°)	3.9 ([-0.2] – 8)	4.3 (1.6 – 8.2)	8 (5.3 – 9.7)	0.002*	Bi vs C < 0.001* Uni vs C 0.002*
Pelvic tilt range (°)	4.2 (3.2 – 5.3)	3.8 (2.9 – 4.7)	3.7 (2.6 – 4.9)	0.078	—
Pelvic obliquity range (°)	8.8 (5.3 – 11.1)	7.4 (4 – 9.8)	7.8 (6.1 – 10)	0.021*	Bi vs Uni 0.005*
Pelvic rotation range (°)	11.9 (9.2 – 13.9)	9.8 (7.1 – 14)	10.8 (7.1 – 15.8)	0.225	—
Peak hip flexion at swing (°)	25.1 (19.1 – 30.3)	25.1 (17 – 30.4)	21 (19 – 31.2)	0.097	—
Peak hip extension (°)	-15.5 ([-18] – [-9.5])	-14.3 ([-19.9] – [-10.7])	-14.6 ([-22] – [-8.4])	0.385	—
Hip sagittal plane range (°)	37.1 (32.7 – 39.3)	39.5 (35.4 – 44)	37.6 (36 – 40.1)	< 0.001*	Bi vs Uni 0.016* Bi vs C 0.009* Uni vs C < 0.001*
Hip frontal plane range (°)	12.5 (8.7 – 14.4)	9.9 (7.9 – 11.6)	12.2 (9.8 – 14.2)	< 0.001*	Bi vs Uni 0.002* Bi vs C < 0.001*
Mean hip rotation at stance (°)	3.7 ([-4] – 13.5)	7.2 (0.9 – 15.6)	4.2 ([-0.6] – 18.5)	0.011*	Bi vs Uni 0.013* Bi vs C 0.004*
Knee frontal plane range (°)	9.7 (7.9 – 11.7)	8.8 (6.4 – 19.1)	8.3 (6.3 – 10.7)	0.262	—
Mean knee valgus at stance (°)	4.9 (2.8 – 7.3)	4.8 (3 – 6.9)	2.8 ([-0.4] – 5.8)	0.002*	Uni vs C (0.005*)
Knee sagittal plane range (°)	56.7 (55 – 60.1)	54.2 (45.7 – 59.3)	57.2 (54.6 – 62.5)	< 0.001*	Bi vs C < 0.001* Uni vs C **0.011**
Mean knee flexion at stance (°)	12.6 (7.7 – 15.3)	8.8 (2.2 – 12.7)	5.2 (1.4 – 7.4)	< 0.001*	Bi vs Uni 0.008* Bi vs C 0.016* Uni vs C < 0.001*
Mean knee flexion at swing (°)	31.6 (30 – 36)	28.5 (24.9 – 32.1)	30.11 (27.8 – 33.6)	< 0.001*	Bi vs Uni < 0.001* Bi vs C 0.008*
Ankle sagittal plane range (°)	24.9 (21.4 – 30.7)	22.4 (18.1 – 27.4)	26.5 (23 – 30)	0.011*	Bi vs C 0.005*
Peak dorsiflexion at stance (°)	14.4 (12.7 – 17.9)	13.9 (11.7 – 18.2)	13.6 (9.8 – 16.2)	0.157	—
Peak dorsiflexion at swing (°)	5 (3.9—7.7)	8.2 (3.7—11)	2.9 ([-2.3]—7.9)	< 0.001*	Bi vs Uni 0.009* Bi vs C < 0.001*
Peak plantar flexion (°)	-10.8 ([-14.8] – [-6.3])	-6.9 ([-13.6] – [-2.2])	-13.7 ([-20.6] – [-8.2])	0.003*	Bi vs C 0.002*

Data are presented as median (IQR). In pair-wise group comparisons, only statistically significant associations are presented

^*^Statistical significance was set at *p* < 0.05

The knee sagittal plane range was significantly lower in the bilateral group than in the control group, with no significant difference between unilateral and bilateral groups. Significant differences were observed in the mean knee flexion at stance among the groups (control group > bilateral group > unilateral group). There was no considerable difference in the mean knee flexion at swing between the unilateral and control groups, but the bilateral group showed lower values than the other groups.

Regarding the ankle sagittal plane range, the bilateral group demonstrated lower values than the control and unilateral groups. The difference only reached statistical significance between bilateral and control groups. Whereas there was no remarkable difference in peak dorsiflexion at stance, peak dorsiflexion at swing significantly differed among the three groups (bilateral group > unilateral group > control group). The bilateral group exhibited lower values than the other groups concerning peak plantarflexion, but the difference only approached statistical significance between bilateral and control groups.

## SKG pattern analysis

**Fig. 3:**
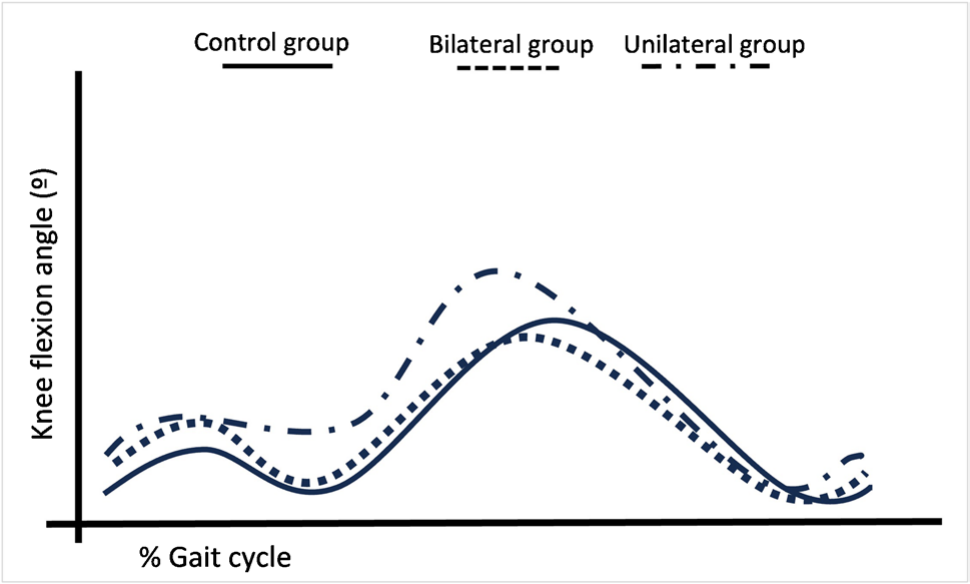
The graph shows the sagittal-plane knee kinematics of each group

Based on the analysis for SKG criteria, three children had borderline SKG status, and two had not-stiff limbs in the unilateral group. In the bilateral group, four children had stiff limbs, and one had borderline SKG status (Table 4). All the SKG parameters significantly differed among the groups in multi-group comparisons (*p* < 0.001 for each parameter). In pair-wise group comparisons, the mean peak knee flexion was significantly lower in the bilateral group (50.12 ± 5.98) than in the control group (54.88 ± 4.75) (*p* < 0.001), with the highest value in the unilateral DDH (58.71 ± 3.26) (*p* < 0.001). In the mean range of knee flexion in early swing, while there was no significant difference between the unilateral (22.8 ± 6.41) and bilateral (23.2 ± 6.92) groups (*p* > 0.05), the parameter was lower in both bilateral and unilateral groups than in the control group (29.32 ± 9.49) (*p* < 0.001). While the total range of knee flexion did not significantly differ between the unilateral (53.56 ± 6.94) and bilateral groups (50.47 ± 7.89) (*p* > 0.05), the parameter was lower in both bilateral (50.47 ± 7.89) (*p* < 0.001) and unilateral (53.56 ± 6.94) groups compared to the control group (58.07 ± 5.18) (*p* < 0.011**)** Table 5. In the mean peak knee flexion time in the swing, a significant difference was observed only between the unilateral (11.5 ± 2.06) and control (12.86 ± 2.71) groups (*p* < 0.001) (Fig. 3).

**Table 4:** The analysis for SKG criteria in bilateral and unilateral DDH groups

	P1) PKF angle (°)		P2) Range of knee flexion between toe-off and PKF (°)		P3) Total range of knee flexion (°)		P4) PKF time in swing (°)		Number of available SKG criteria	SKG status
	Mean	SD	Mean	SD	Mean	SD	Mean	SD		
Bilateral group										
Patient 1	**51.28**	1.82	**19.48**	4.55	**42.70**	2.19	11.92	1.83	3	stiff
Patient 2	**46.49**	6.17	**19.84**	6.31	**48.78**	8.32	11.17	1.47	3	stiff
Patient 3	**51.45**	4.49	**25.22**	6.97	**51.21**	1.93	**13.08**	1.93	4	stiff
Patient 4	**45.59**	5.20	**29.23**	6.13	58.62	1.31	**13.58**	1.31	3	stiff
Patient 5	55.80	5.29	**22.26**	6.17	**51.07**	1.73	11.42	1.73	2	borderline
Unilateral group										
Patient 1	57.73	3.21	**16.35**	3.81	**42.99**	3.28	9.83	0.98	2	borderline
Patient 2	57.96	1.18	**20.61**	5.26	**50.53**	2.61	11.17	1.94	2	borderline
Patient 3	56.70	1.46	**20.83**	4.07	**55.29**	3.78	11.00	2.19	2	borderline
Patient 4	57.17	1.00	**25.87**	3.91	60.69	1.78	11.33	1.03	1	not stiff
Patient 5	64.01	1.90	30.38	4.90	58.33	2.79	**14.17**	1.33	1	not stiff
Control group										
	54.88	4.75	29.32	9.49	58.07	5.18	12.86	2.71		

All SKG parameter values are given as mean and SD

Bold shows that the t-test found value as statistically different from normal, *p* < 0.05

The average normal value for each SKG parameter was calculated based on the data obtained from the control group

*SKG* stiff-knee gait, *DDH* developmental dysplasia of the hip, *PKF* peak knee flexion, *SD* standard deviation

**Table 5:** Comparative result of SKG parameters among the groups

Parameters	Unilateral group (*n* = 5)	Bilateral group (*n* = 5)	Control group (*n* = 10)	Multi-group comparison (*P* values)	Pair-wise group comparison (*P* values)
P1) peak knee flexion angle (°)	57.8 (56.2 – 61.0)	50.1 (47.1 – 52.6)	53.5 (51.7 – 59.0)	< 0.001*	Bi vs Uni ** < 0.001** Bi vs C ** < 0.001** Uni vs C ** < 0.001**
P2) range of knee flexion in early swing (°)	22.4 (18 – 36.7)	22.0 (18.8 – 28.8)	30.6 (22.7 – 35.8)	< 0.001*	Bi vs C ** < 0.001** Uni vs C ** < 0.001**
P3) total range of knee flexion (°)	54.3 (48.2 – 59.5)	49.7 (44.7 – 57.9)	57.3 (54.1 – 62.6)	< 0.001*	Bi vs C ** < 0.001** Uni vs C **0.011**
P4) peak knee flexion time in the swing	11 (10 – 13)	12 (11 – 13.8)	14 (11.3 – 15)	< 0.001*	Uni vs C **(< 0.001)**

Data are presented as median (IQR). In pair-wise group comparisons, only statistically significant associations are presented.

*Statistical significance was set at *p* < 0.05

## Kinetic gait analysis

Table 6 depicts the results of the kinetic gait analysis. Most kinetic gait parameters were not statistically different between groups. However, a significantly lower peak hip abduction moment (30–60% of the gait cycle) and lower peak knee flexion moment (30–60% of the gait cycle) were found in the unilateral group (-0.48 ± 0.2; **-**0.24 ± 0.19) than in the control group (-0.78 ± 0.32, *p* < 0.001; -0.45 ± 0.15, *p* = 0.002), respectively. These parameters of the unilateral group were also lower than those of the bilateral group, but the difference reached no statistical significance.

**Table 6:** Kinetic gait parameters

Variables	Unilateral group (*n* = 5)	Bilateral group (*n* = 5)	Control group (*n* = 10)	Multi-group comparison *(P *values*)*	Pairwise group comparison *(P *values*)*
Peak hip extension moment at swing (Nm/kg)	0.1 (0.09 – 0.2)	0.2 (0.1 – 0.2)	0.2 (0.1 – 0.3)	0.127	-
Peak hip flexion moment (Nm/kg)	-0.7 ([-0.8] – [-0.5])	-0.6 ([-0.9] – [-0.5])	-0.8 ([-0.9] – [-0.6])	0.232	-
Peak hip power absorption (30–60%) (Nm/kg)	-0.4 ([-0.6] – [-0.3])	-0.5 ([-0.7] – [-0.4])	-0.8 ([-0.9] – [-0.5])	0.004*	**Uni vs C (0.001)**
Peak hip power generation (30–60%) (wt/kg)	0.3 (0.2 – 0.4)	0.4 (0.3 – 0.6)	0.4 (0.2 – 0.7)	0.139	-
Peak hip abduction moment (0–30%) (Nm/kg)	0.6 (0.5 – 0.7)	0.6 (0.4 – 0.7)	0.7 (0.5 – 0.8)	0.162	-
Peak hip abduction moment (30–60%) (Nm/kg)	0.5 (0.5 – 0.6)	0.5 (0.3 – 0.7)	0.6 (0.4 – 0.8)	0.195	-
Peak knee extension moment (30–60%)(Nm/kg)	0.1 (0.03 – 0.2)	0.1 (0.1 – 0.2)	0.07 (0.05 – 0.1)	0.636	-
Peak knee flexion moment (30–60%) (Nm/kg)	-0.22 ([-0.3] – [0.1])	-0.3 ([-0.5] – [-0.2])	-0.5 ([-0.5] – [-0.4])	< 0.005*	**Uni vs C** **0.002**
Peak ankle plantar flexion moment (Nm/kg)	1.2 (1.2 – 1.5)	1.4 (1 – 1.5)	1.4 (1.2 – 1.5)	0.573	-
Peak ankle power generation (wt/kg)	3.6 (2.3 – 4.4)	3.3 (2.7 – 4.1)	3.3 (3 – 3.9)	0.888	-

Data are presented as median (IQR). Bold data indicate statistically significant values

In pair-wise group comparisons, only statistically significant associations are presented

^*^Statistical significance was set at *p* < 0.05

## Discussion

Tenotomies of the iliopsoas and adductor longus are important steps during open reduction of a developmental dislocated hip that have a direct impact on obtaining and maintaining a stable, concentrically reduced hip joint. Nonetheless, the literature remains scarce and inconsistent as to whether these tenotomies cause negative effects on gait patterns at the long-term following MOR in the management of children with DDH [7, 12, 13, 15]. The main findings of the current study are that there are notable deviations in the gait patterns of children with DDH treated by MOR at long-term follow-up compared to healthy children’s gait patterns.

When analyzing the results of this study, all the spatiotemporal gait parameters differed significantly among the groups except for the swing time. The stance and double support times of the unilateral and bilateral DDH groups were comparable based on pair-wise comparisons, but those were shorter in both DDH groups compared to the healthy group. Additionally, both DDH groups exhibited shorter strides and step lengths along with faster cadence. Of note, the differences in length and cadence values only reached statistical significance for the bilateral group, not the unilateral group. However, the trend favored the decrease in these values of children with unilateral DDH. It can be assumed that the reduction in stance and support times result from the decrease in stride and step lengths. Given the similar gait velocity of all groups, children with DDH seems to increase the cadence to tolerate these alterations. These spatiotemporal gait deviations could be attributed to the long-term negative effect of *impaired functions of the iliopsoas and adductor muscles* following MOR. Particularly, these alterations are more pronounced in children undergoing bilateral DDH, reinforcing our first hypothesis that children with DDH treated by MOR could develop significant gait deviations at the long-term compared to healthy controls due to impaired functions of the iliopsoas and adductor muscles.

Other important findings of the current study supporting the first hypothesis include detectable changes in kinematic and kinetic gait parameters. At normal walking velocity, both DDH groups exhibited decreased anterior pelvic tilt compared to the healthy controls, and children with unilateral DDH demonstrated the highest amount of pelvic obliquity. Impaired functions of the iliopsoas and adductor muscles following MOR may have resulted in a posterior pelvic tilt in a similar pelvic tilt range, regardless of unilateral or bilateral application. Furthermore, it is well-known that bilateral control of the hip abductor and adductor muscles is essential for pelvic stability [21]. Thus, the increased pelvic obliquity in children with unilateral DDH could be caused by unilateral adductor muscle weakness since there is no compensatory force on the contralateral side. In addition, there were no significant changes in the range of pelvic rotation between groups, as pelvic rotation occurs primarily in the horizontal plane, whereas the iliopsoas and adductor muscles produce movements predominantly in the sagittal and frontal planes, respectively. Therefore, these findings can be interpreted as supporting the second hypothesis that MOR could negatively affect pelvic motion during the gait cycle of the affected extremity due to impaired functions of the iliopsoas and adductor muscles.

An interesting result from the study included SKG analyses of the study participants. SKG is a common gait disorder in ambulatory children with cerebral palsy, characterized by diminished PKF and total knee ROM as well as delayed time to the PKF [22]. The main cause of SKG is the over-activity of the rectus femoris muscle in the swing or pre-swing phase of gait, resulting in tripping during the swing phase and a greater expenditure of energy when walking [11, 19]. Moreover, in a recent study by Akalan et al. investigating the relationship between iliopsoas muscle weakness and SKG during walking in healthy participants, the authors suggested that any treatment approach that lessens hip flexion during gait by weakening the iliopsoas muscle could produce a SKG pattern along with a slower gait velocity [11]. Based on the existing literature, we hypothesized that iliopsoas tenotomy could influence swing-phase knee flexion and contribute to SKG in children treated by MOR. Our results supported this hypothesis, as four children undergoing bilateral MOR exhibited SKG pattern based on the definition of Goldberg et al. Nonetheless, no SKG was observed in the long-term following unilateral MOR. Suppose unilateral iliopsoas tenotomy does not result in a stiff knee gait pattern in the long term. In that case, it might be due to preserving some degree of balance in muscle function and joint dynamics. Unilateral procedures allow for better compensation and adaptation by the musculoskeletal system, reducing the likelihood of gait abnormalities.

According to our literature review, very little research has been conducted on long-term residual gait changes in children with DDH who underwent MOR. In one study, Ömeroğlu et al. (2008) [15] investigated the long-term effects of posteromedial soft tissue release, including sectioning of the adductor longus and iliopsoas tendons, on 3-D quantitative gait analysis of ten patients (mean age, 8.1 years) with unilateral DDH surgically treated under the age of 18 months. Similar to our study design, the authors compared those data with healthy controls. They concluded that despite mild gait deviations compared to healthy ones, the posteromedial soft tissue surgery did not cause significant alterations in the gait analysis at midterm follow-up in DDH. In another study, Chang et al. (2012) [13] performed quantitative gait analysis on eleven females (mean age: 10.6 ± 1.0 years) in whom open reduction with Pemberton’s osteotomy was applied for unilateral DDH at 1.6 ± 0.5 years of age and eleven age-matched healthy controls. In the Pemberton group, an asymmetrical gait pattern was identified, including increased knee flexion and ankle dorsiflexion in the affected limb, hiking at the affected side, and rotation towards the unaffected side of the pelvis. The authors inferred that those gait deviations could be due to the altered pelvic motions to reduce loads of those muscles in the affected hip often involved during the surgery, despite the possibility of increasing harmful loading rates at both hip joints. Findings from the present study supported the previous studies, indicating that MOR could adversely affect pelvic motion during gait, secondary to impaired functions of *the iliopsoas and adductor muscle.* Unlike previous research [13, 15], the current study contributes to the existing literature by examining the relationship between MOR and SKG, comparing bilateral DDH to healthy controls in addition to unilateral cases, with a longer follow-up period.

The present study has several limitations, and the study results and conclusions should be interpreted keeping these in perspective. The main limitation was the small sample size, which reduced the generalizability of the findings. Another limitation was its retrospective design, which contributed to selection bias. It’s also important to note that this is a nonrandomized, observational study, meaning there might be several hidden, confounding factors and potential selection biases in the data. Finally, the study has solely focused on analyzing gait data to test hypotheses. However, there is a dearth of clinical data in this investigation, which includes objective assessments such as muscular strength and electromyography, as well as radiological evaluations like magnetic resonance imaging findings. Future prospective studies are needed to validate our findings in a larger cohort for better generalization. Nevertheless, the current study is one of the few studies dealing with gait patterns and deviations at long-term follow-up in children who underwent MOR. Furthermore, this is the first study to focus on the development of SKG in this specific cohort.

## Conclusion

This study has revealed notable deviations in gait patterns of children with DDH treated by MOR at long-term follow-up compared to healthy children’s gait. MOR could negatively affect pelvic motion during gait due to impaired functions of *the iliopsoas and adductor muscles*, and SKG can be encountered secondary to iliopsoas weakness. Physical rehabilitation should be given for stiff knee and pelvic asymmetry following MOR in managing DDH.

## Data Availability

Not applicable.
