# Correction to: Residual gait deviations in children treated by medial open reduction for developmental dysplasia of the hip at long‑term follow‑up: a comparison with healthy controls

**DOI:** 10.1007/s00264-024-06285-3

**Published:** 2024-08-27

**Authors:** Mehmet Demirel, Halenur Evrendilek, N. Ekin Akalan, Fuat Bilgili, Emre Meriç, Shavkat Kuchimov, Kübra Önerge

**Affiliations:** 1 Department of Orthopaedics and Traumatology, İstanbul School of Medicine, İstanbul University, Istanbul, Turkey; 2 Physiotherapy and Rehabilitation Department, Faculty of Health Sciences, İstanbul Kültür University, Istanbul, Turkey; 3 Institute of Biomedical Engineering, Boğaziçi University, Istanbul, Turkey

**Correction to: International Orthopaedics**

10.1007/s00264-024-06263-9

The correct affiliation of Kübra Önerge should be affiliation 2:

Physiotherapy and Rehabilitation Department, Faculty of Health Sciences, İstanbul Kültür University, Istanbul, Turkey.

and not affiliation 3.

The original article has been corrected.

